# Supplementary material for: Denoising the Denoisers: an independent evaluation of microbiome sequence error-correction approaches
Source: PeerJ. 2018 Aug 8;6:e5364. doi: 10.7717/peerj.5364 (PMC6087418; doi:10.7717/peerj.5364)
Supplement: Figure S1 — ASVs/OTUs output by each sequence processing method on the Human Microbiome Project mock community were compared to expected sequences using BLASTN. Sequence identity were then binned to within a 1% difference in sequence identity. ALL sequences with identity below 75% were binned together. (A) DADA2; (B) Deblur; (C) UNOISE3; (D) Open reference 97% OTU clustering. [file peerj-06-5364-s001.pdf]

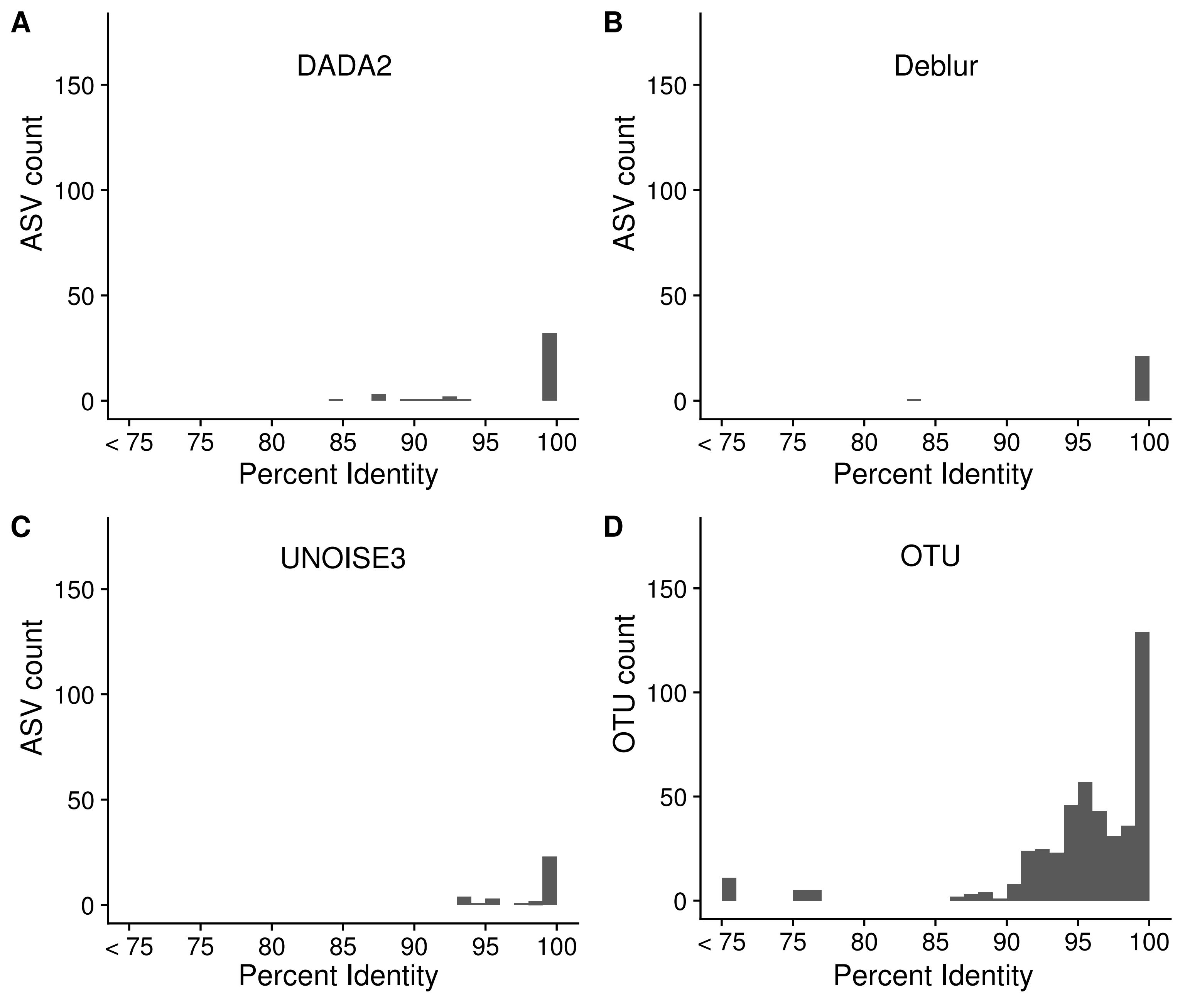
A
DADA2
ASV count
150
100
50
0
< 75
75
80
85
90
95
100
Percent Identity
B
Deblur
ASV count
150
100
50
0
< 75
75
80
85
90
95
100
Percent Identity
C
UNOISE3
ASV count
150
100
50
0
< 75
75
80
85
90
95
100
Percent Identity
D
OTU
OTU count
150
100
50
0
< 75
75
80
85
90
95
100
Percent Identity

Supplemental Figure 1: Human Microbiome Project mock community sequence similarity

ASVs/OTUs output by each sequence processing method on the Human Microbiome Project mock community were compared to the expected sequences from the mock community using BLASTN. Sequences were then binned together based on their difference in sequence identity to the expected sequences. ALL sequences with identity below 75% were binned together. A) DADA2; B) Deblur; C) UNOISE3; D) Open reference 97% OTU clustering
