# Supplementary material for: Denoising the Denoisers: an independent evaluation of microbiome sequence error-correction approaches
Source: PeerJ. 2018 Aug 8;6:e5364. doi: 10.7717/peerj.5364 (PMC6087418; doi:10.7717/peerj.5364)
Supplement: Figure S4 — Amplicon sequence variants/operational taxonomic units (ASVs/OTUs) were run through an abundance filtering at 0.1% (HMP, Fungal, Zymomock) or 0.0004% (Extreme) remaining ASVs/OTUs were compared to a database of full-length amplicon sequences for just the microbes supposedly in the community (“Expected”) and against the full SILVA or ITS databases (“Database”) using BLASTN at 97% and 100% identity cut-offs. “Unmatched” sequences did not match an expected sequence or the SILVA/ITS databases at 97% identity or greater. Dotted lines indicate the total number of ASVs/OTUs expected, accounting for 16S copy variation within genomes. Note that the y-axis for open reference OTU clustering is different than the y axis on the denoising methods. (A) Human Microbiome Project equal abundance mock community; (B) Extreme dataset; (C) Fungal ITS1 mock community; (D) Zymomock community. [file peerj-06-5364-s004.pdf]

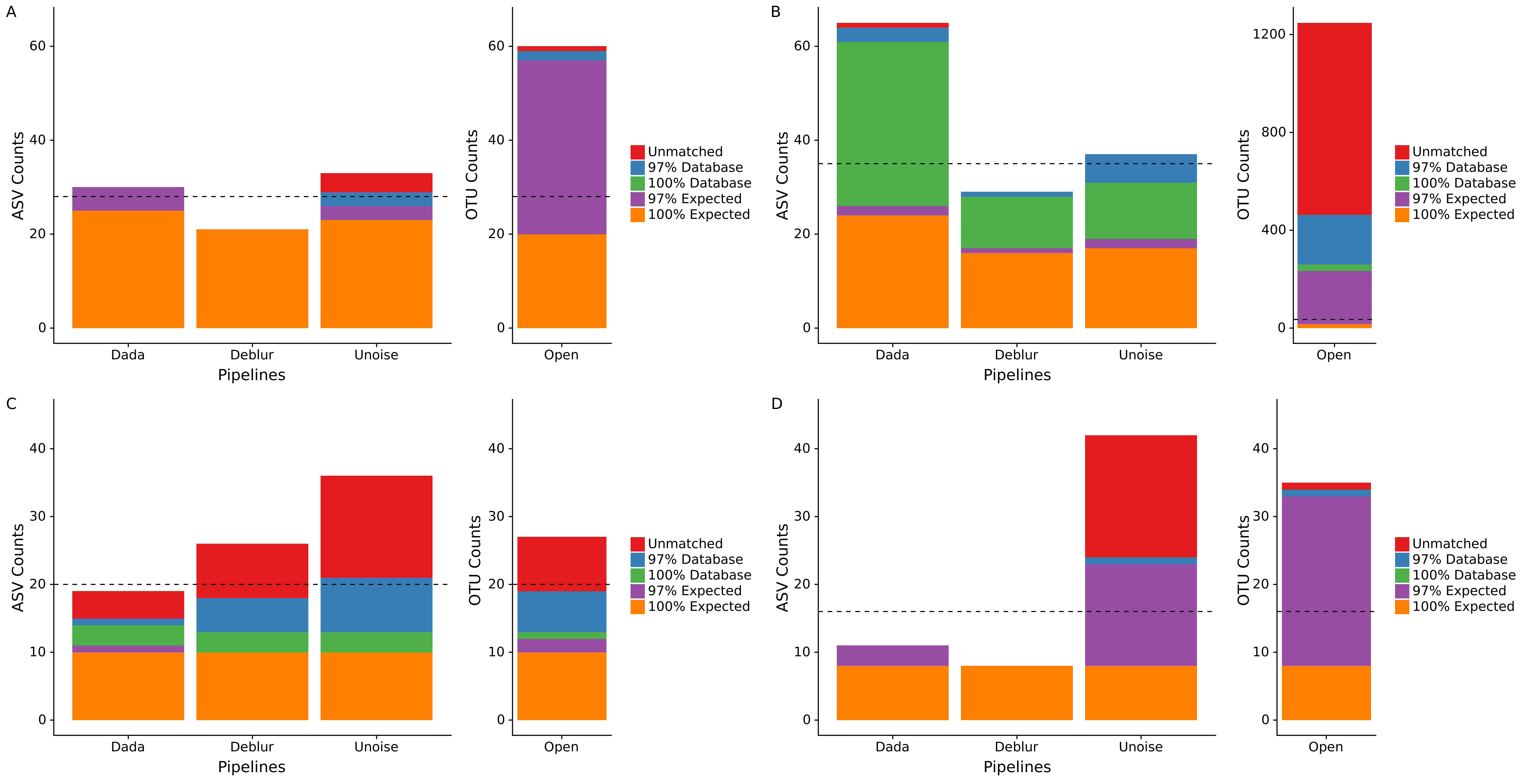

A
B
C
D
ASV Counts
OTU Counts
Pipelines
Dada
Deblur
Unoise
Open
Unmatched
97% Database
100% Database
97% Expected
100% Expected

Supplemental Figure 4: Total number of ASVs/OTUs identified by each sequence processing method for four different mock communities after removal of low abundance ASVs/OTUs reveals large shift in number of OTUs

Amplicon sequence variants/operational taxonomic units (ASVs/OTUs) were filtered at an abundance of 0.1% (HMP, Fungal, Zymomock) or 0.0004% (Extreme) remaining ASVs/OTUs were compared to a database of full-length amplicon sequences for just the microbes supposedly in the community (“Expected”) and against the full SILVA or ITS databases (“Database”) using BLASTN at 97% and 100% identity cut-offs. “Unmatched” sequences did not match an expected sequence or the SILVA/ITS databases at 97% identity or greater. Dotted lines indicate the total number of ASVs/OTUs expected, accounting for 16S copy variation within genomes. Note that the y-axis for open reference OTU clustering is different than the y axis on the denoising pipelines. A) Human Microbiome Project equal abundance mock community; B) Extreme dataset; C) Fungal ITS1 mock community; D) Zymomock community
