# Supplementary material for: Denoising the Denoisers: an independent evaluation of microbiome sequence error-correction approaches
Source: PeerJ. 2018 Aug 8;6:e5364. doi: 10.7717/peerj.5364 (PMC6087418; doi:10.7717/peerj.5364)
Supplement: Figure S5 — (A–C) The Bray–Curtis dissimilarity distances between the same biological samples based on ASVs/OTUs outputted by each of the different sequence processing methods on the soil, human associated, and Exercise datasets respectively. (D–F) Non-metric multidimensional scaling plots of all the samples in the soil, human associated, and Exercise datasets respectively. The four different sample profiles generated for each biological sample are colour-coded and are joined by an interconnecting line. [file peerj-06-5364-s005.pdf]

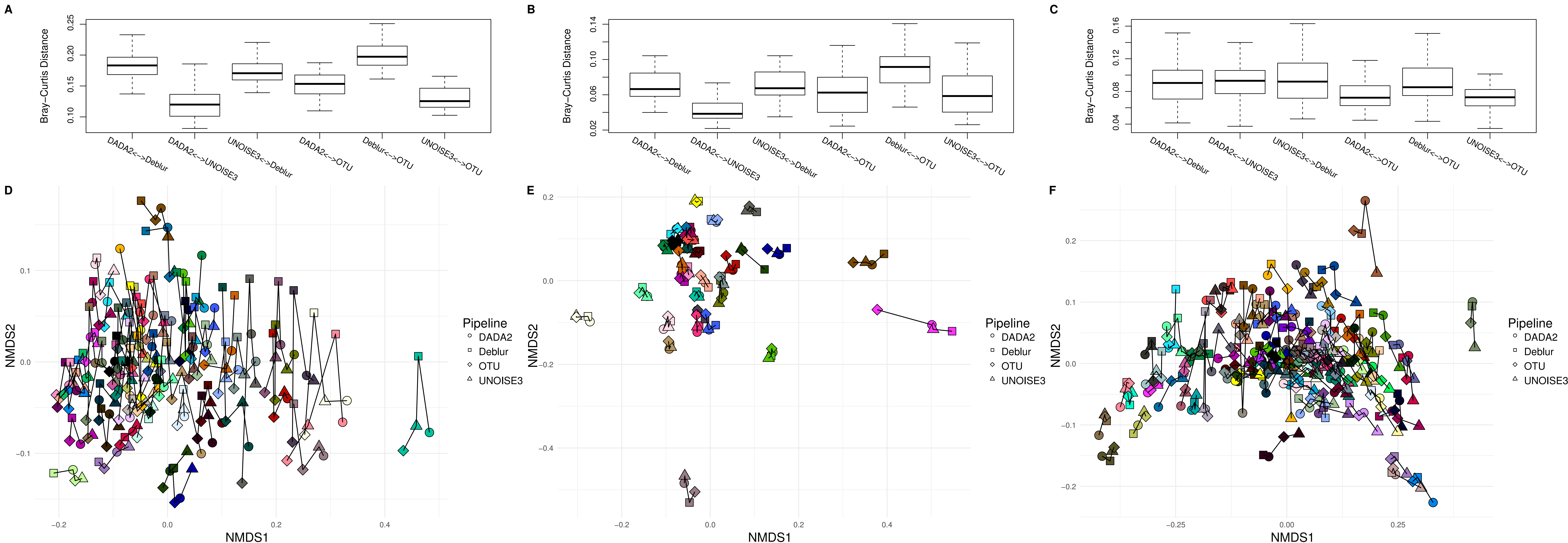

A
B
C
Bray-Curtis Distance
DADA2<-->Deblur
DADA2<-->UNOISE3
UNOISE3<-->Deblur
DADA2<-->OTU
Deblur<-->OTU
UNOISE3<-->OTU
D
E
F
NMDS1
NMDS2
Pipeline
DADA2
Deblur
OTU
UNOISE3

Supplemental Figure 5: Bray-Curtis dissimilarity intra-sample distances between sequencing processing methods based on three real datasets reveals relatively similar biological profiles.

A-C) The Bray-Curtis dissimilarity distances between the same biological samples based on ASVs/OTUs outputted by each of the different sequence processing methods on the soil, human associated, and Exercise datasets respectively. D-F) Non-metric multidimensional scaling plots of all the samples in the soil, human associated, and Exercise datasets respectively. The four different sample profiles generated for each biological sample are colour-coded and are joined by an interconnecting line.
