# Supplementary material for: Denoising the Denoisers: an independent evaluation of microbiome sequence error-correction approaches
Source: PeerJ. 2018 Aug 8;6:e5364. doi: 10.7717/peerj.5364 (PMC6087418; doi:10.7717/peerj.5364)
Supplement: Figure S6 — (A–C) The unweighted UniFrac distances between the same biological samples based on ASVs/OTUs outputted by each of the different sequence processing methods on the soil, human associated and Exercise datasets respectively. (D–F) Principal coordinates analysis of the unweighted UniFrac distances of all the samples in the soil, human associated, and Exercise datasets respectively. The four different sample profiles generated for each biological sample are colour-coded and are joined by an interconnecting line. [file peerj-06-5364-s006.pdf]

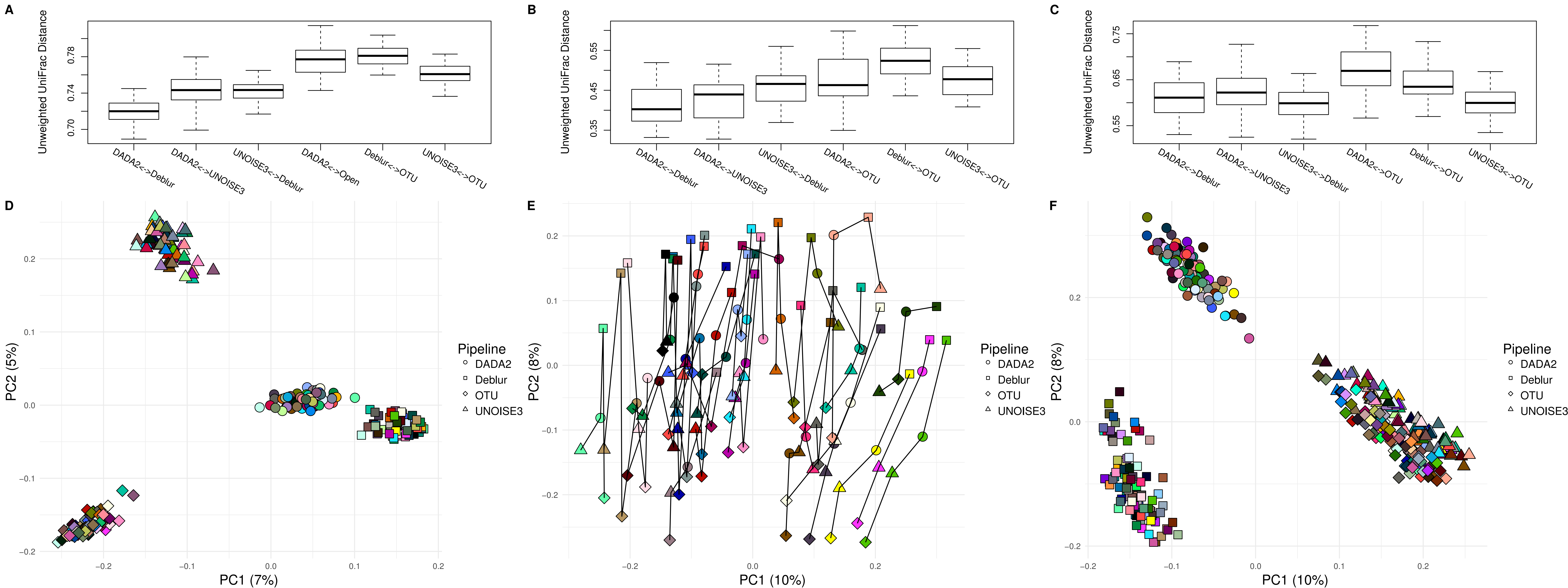

A
Unweighted UniFrac Distance
0.70
0.74
0.78
DADA2<->Deblur
DADA2<->UNOISE3
UNOISE3<->Deblur
DADA2<->Open
Deblur<->OTU
UNOISE3<->OTU
B
Unweighted UniFrac Distance
0.35
0.45
0.55
DADA2<->Deblur
DADA2<->UNOISE3
UNOISE3<->Deblur
DADA2<->OTU
Deblur<->OTU
UNOISE3<->OTU
C
Unweighted UniFrac Distance
0.55
0.65
0.75
DADA2<->Deblur
DADA2<->UNOISE3
UNOISE3<->Deblur
DADA2<->OTU
Deblur<->OTU
UNOISE3<->OTU
D
PC2 (5%)
PC1 (7%)
Pipeline
DADA2
Deblur
OTU
UNOISE3
E
PC2 (8%)
PC1 (10%)
Pipeline
DADA2
Deblur
OTU
UNOISE3
F
PC2 (8%)
PC1 (10%)
Pipeline
DADA2
Deblur
OTU
UNOISE3

Supplemental Figure 6: Unweighted UniFrac intra-sample distances between sequence processing methods based on three real datasets reveal highly different biological profiles.

A-C) The unweighted UniFrac distances between the same biological samples based on ASVs/OTUs outputted by each of the different sequence processing methods on the soil, human associated, and Exercise datasets respectively. D-F) Principal coordinates analysis of the unweighted UniFrac distances of all the samples in the soil, human associated, and Exercise datasets respectively. The four different sample profiles generated for each biological sample are colour-coded and are joined by an interconnecting line.
