# Supplementary material for: Denoising the Denoisers: an independent evaluation of microbiome sequence error-correction approaches
Source: PeerJ. 2018 Aug 8;6:e5364. doi: 10.7717/peerj.5364 (PMC6087418; doi:10.7717/peerj.5364)
Supplement: Figure S7 — (A) The weighted UniFrac intra-sample distances between the same biological samples based on ASVs/OTUs outputted by each sequence processing method and either Deblur or Deblur without positive filtering. (B) The unweighted UniFrac intra-sample distances between the same biological samples based on ASVs/OTUs outputted by each sequence processing method and either Deblur or Deblur without positive filtering. (C) The Bray–Curtis dissimilarity intra-sample distances between the same biological samples based on ASVs/OTUs outputted by each sequence processing method and either Deblur or Deblur without positive filtering. [file peerj-06-5364-s007.pdf]

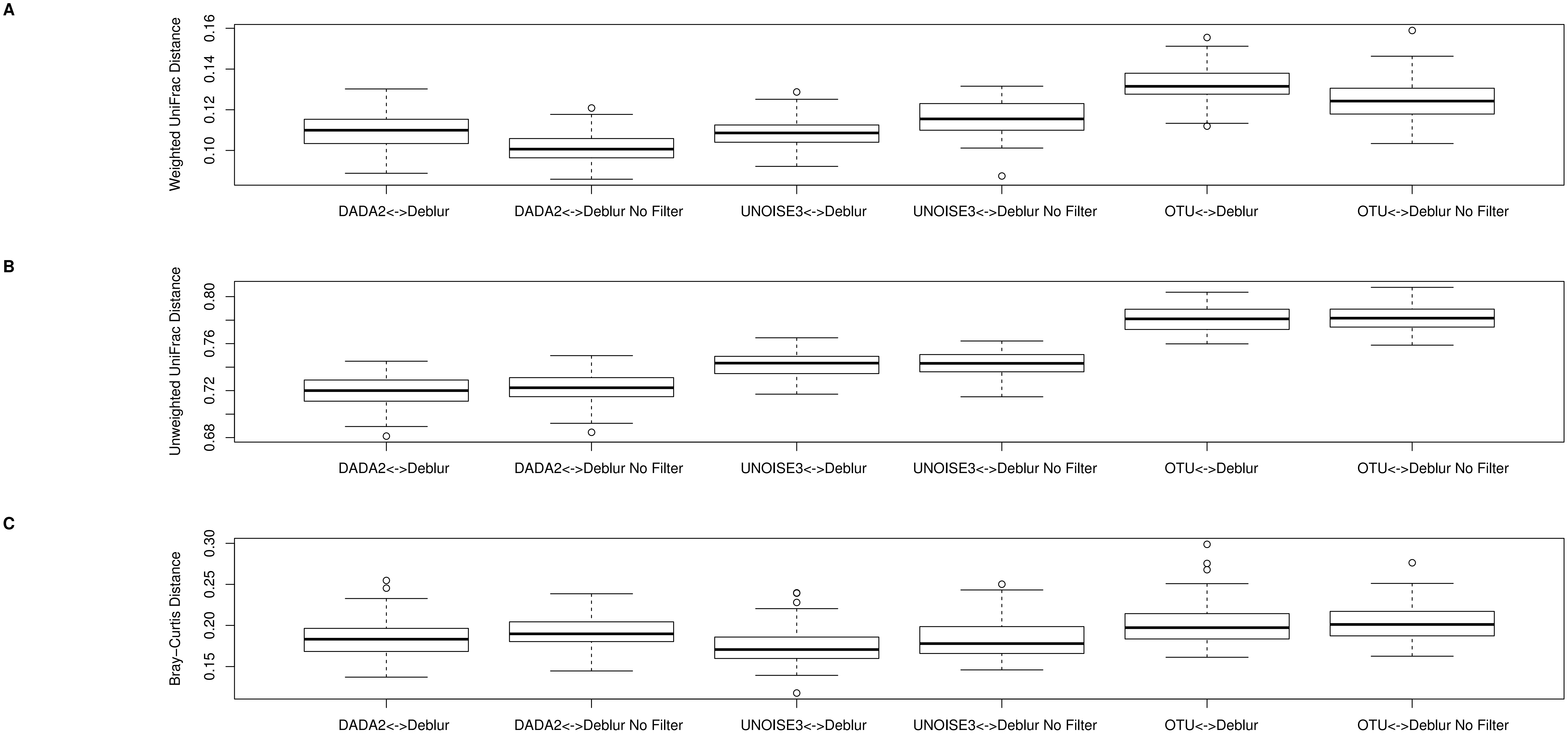
A
Weighted UniFrac Distance
0.10
0.12
0.14
0.16
DADA2<->Deblur
DADA2<->Deblur No Filter
UNOISE3<->Deblur
UNOISE3<->Deblur No Filter
OTU<->Deblur
OTU<->Deblur No Filter
B
Unweighted UniFrac Distance
0.68
0.72
0.76
0.80
DADA2<->Deblur
DADA2<->Deblur No Filter
UNOISE3<->Deblur
UNOISE3<->Deblur No Filter
OTU<->Deblur
OTU<->Deblur No Filter
C
Bray–Curtis Distance
0.15
0.20
0.25
0.30
DADA2<->Deblur
DADA2<->Deblur No Filter
UNOISE3<->Deblur
UNOISE3<->Deblur No Filter
OTU<->Deblur
OTU<->Deblur No Filter

Supplemental Figure 7: Comparing Beta Diversity metrics between Deblur with and without positive filtering reveals little difference between the two based on beta diversity metrics

A) The weighted UniFrac intra-sample distances between the same biological samples based on ASVs/OTUs outputted by each sequence processing method and either Deblur or Deblur without positive filtering. B) The unweighted UniFrac intra-sample distances between the same biological samples based on ASVs/OTUs outputted by each sequence processing method and either Deblur or Deblur without positive filtering. C) The Bray-Curtis dissimilarity intra-sample distances between the same biological samples based on ASVs/OTUs outputted by each sequence processing method and either Deblur or Deblur without positive filtering
