# Supplementary material for: Denoising the Denoisers: an independent evaluation of microbiome sequence error-correction approaches
Source: PeerJ. 2018 Aug 8;6:e5364. doi: 10.7717/peerj.5364 (PMC6087418; doi:10.7717/peerj.5364)
Supplement: Figure S8 — Pairwise scatterplots of the number of ASVs/OTUs found in each biological sample with two different sequence processing methods on each axis. The R value represents the rho correlation between the two different processing methods. [file peerj-06-5364-s008.pdf]

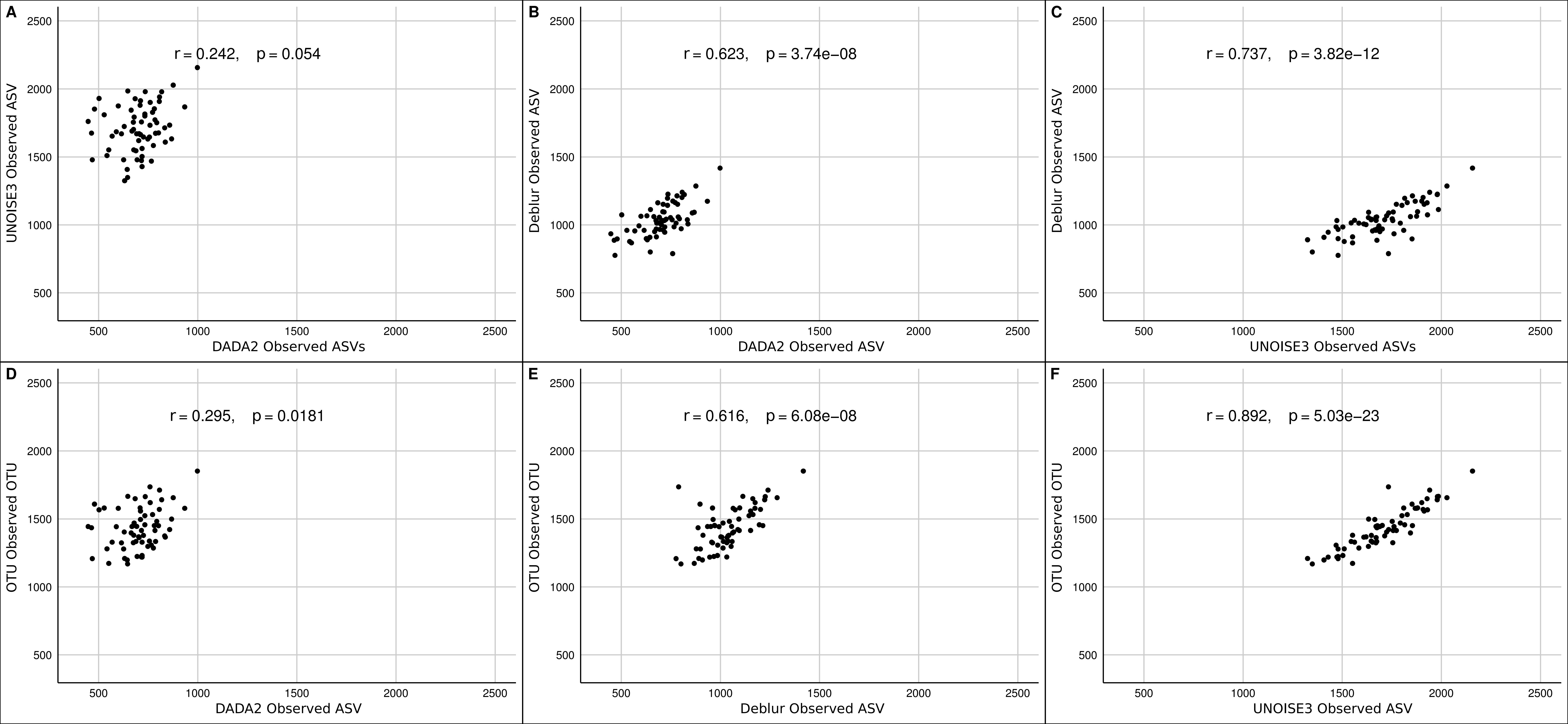
A
r = 0.242, p = 0.054
UNOISE3 Observed ASV
DADA2 Observed ASVs
B
r = 0.623, p = 3.74e−08
Deblur Observed ASV
DADA2 Observed ASV
C
r = 0.737, p = 3.82e−12
Deblur Observed ASV
UNOISE3 Observed ASVs
D
r = 0.295, p = 0.0181
OTU Observed OTU
DADA2 Observed ASV
E
r = 0.616, p = 6.08e−08
OTU Observed OTU
Deblur Observed ASV
F
r = 0.892, p = 5.03e−23
OTU Observed OTU
UNOISE3 Observed ASV
500
1000
1500
2000
2500

Supplemental Figure 8: Scatterplots of ASVs/OTUs found in each sample between the different sequence processing methods on the soil dataset.

Pairwise scatterplots of the number of ASVs/OTUs found in each biological sample with two different sequence processing methods on each axis. The R value represents the rho correlation between the two different processing methods.
