# Supplementary material for: Denoising the Denoisers: an independent evaluation of microbiome sequence error-correction approaches
Source: PeerJ. 2018 Aug 8;6:e5364. doi: 10.7717/peerj.5364 (PMC6087418; doi:10.7717/peerj.5364)
Supplement: Figure S9 — Pairwise scatterplots of the number of ASVs/OTUs found in each biological sample with two different sequence processing methods on each axis. The R value represents the rho correlation between the two different processing methods. [file peerj-06-5364-s009.pdf]

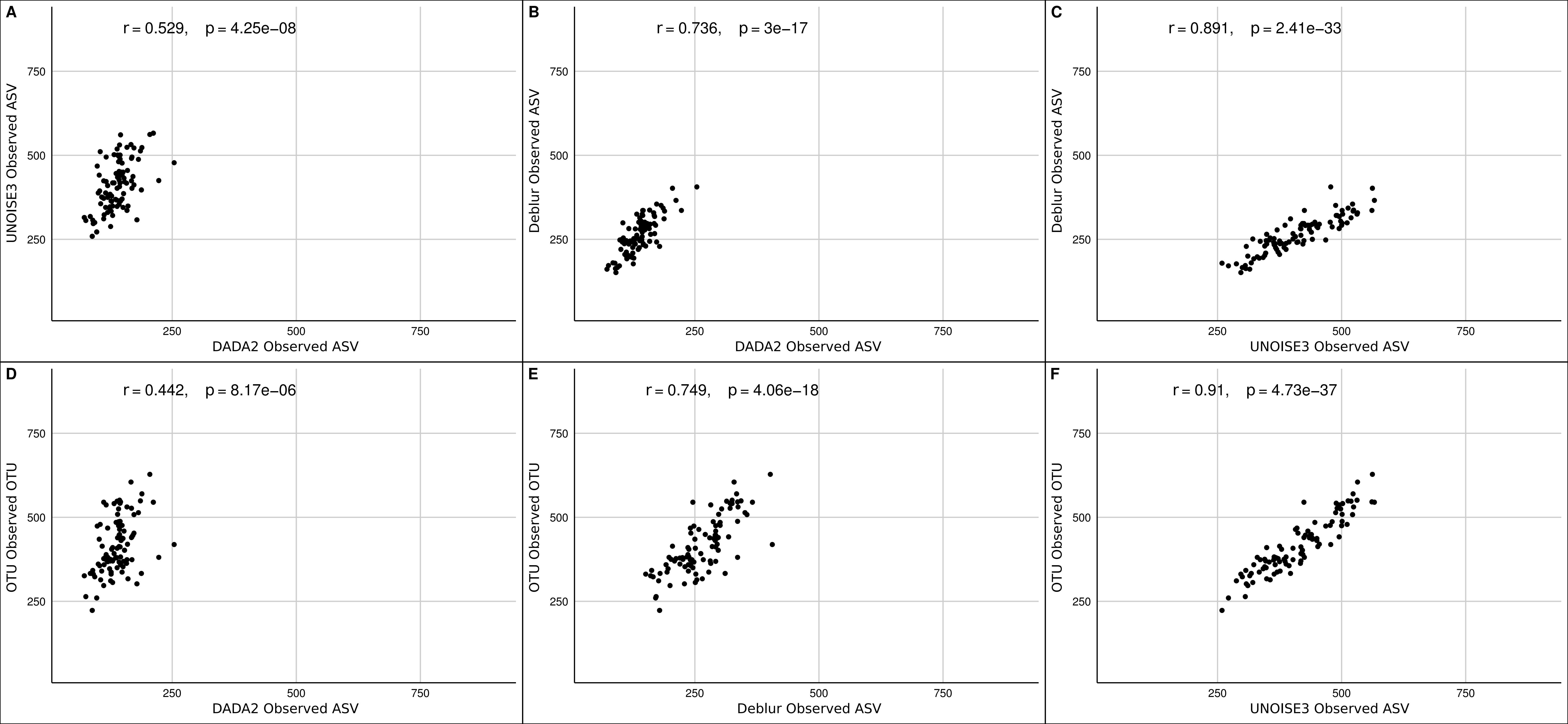

A
r = 0.529, p = 4.25e−08
UNOISE3 Observed ASV
DADA2 Observed ASV
B
r = 0.736, p = 3e−17
Deblur Observed ASV
DADA2 Observed ASV
C
r = 0.891, p = 2.41e−33
Deblur Observed ASV
UNOISE3 Observed ASV
D
r = 0.442, p = 8.17e−06
OTU Observed OTU
DADA2 Observed ASV
E
r = 0.749, p = 4.06e−18
OTU Observed OTU
Deblur Observed ASV
F
r = 0.91, p = 4.73e−37
OTU Observed OTU
UNOISE3 Observed ASV
250
500
750

Supplemental Figure 9: Scatterplots of ASVs/OTUs found in each sample between the different sequence processing methods on the Exercise dataset.

Pairwise scatterplots of the number of ASVs/OTUs found in each biological sample with two different sequence processing methods on each axis. The R value represents the rho correlation between the two different processing methods.
