# Supplementary material for: Denoising the Denoisers: an independent evaluation of microbiome sequence error-correction approaches
Source: PeerJ. 2018 Aug 8;6:e5364. doi: 10.7717/peerj.5364 (PMC6087418; doi:10.7717/peerj.5364)
Supplement: Figure S10 — Pairwise scatterplots of the number of ASVs/OTUs found in each biological sample with two different sequence processing methods on each axis. The R value represents the rho correlation between the two different processing methods. [file peerj-06-5364-s010.pdf]

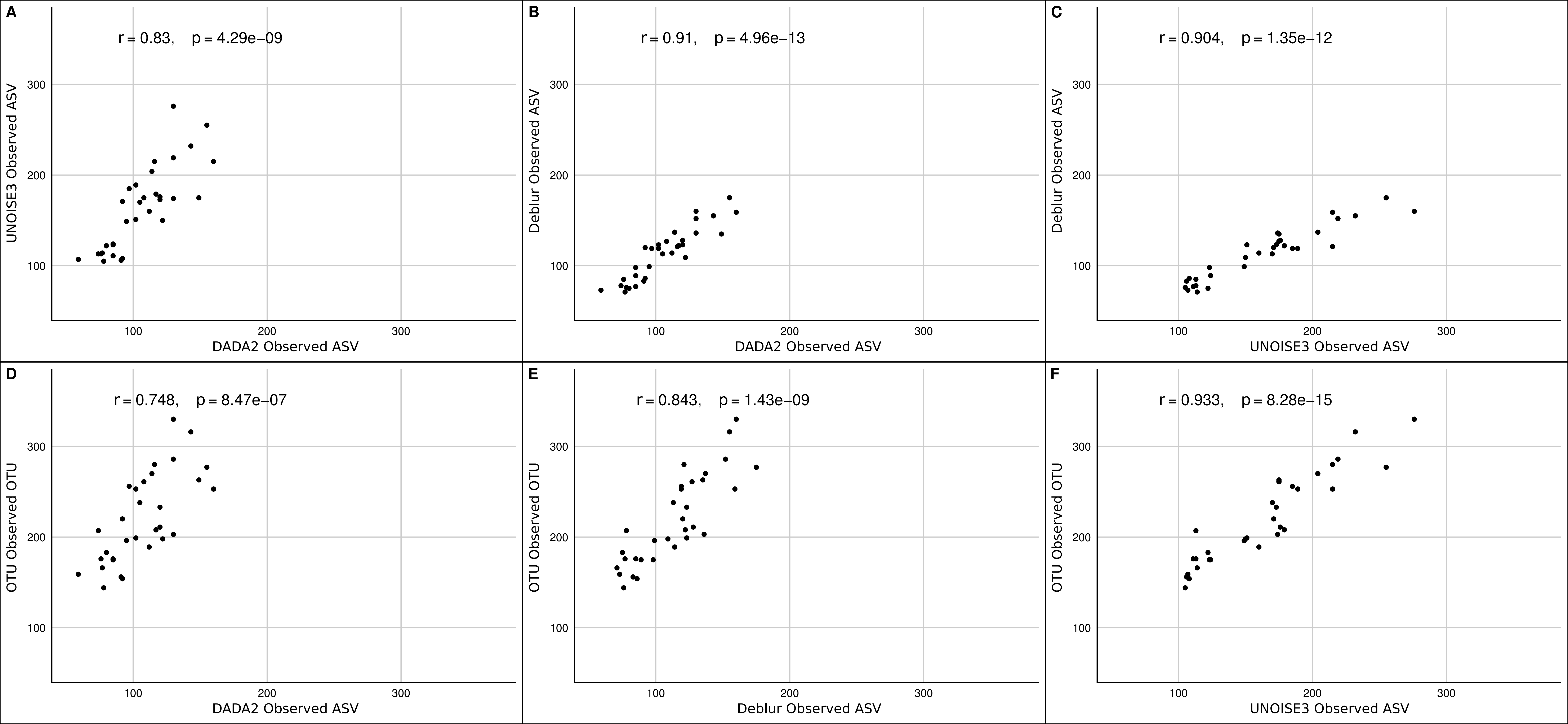

A
r = 0.83, p = 4.29e−09
UNOISE3 Observed ASV
DADA2 Observed ASV
B
r = 0.91, p = 4.96e−13
Deblur Observed ASV
DADA2 Observed ASV
C
r = 0.904, p = 1.35e−12
Deblur Observed ASV
UNOISE3 Observed ASV
D
r = 0.748, p = 8.47e−07
OTU Observed OTU
DADA2 Observed ASV
E
r = 0.843, p = 1.43e−09
OTU Observed OTU
Deblur Observed ASV
F
r = 0.933, p = 8.28e−15
OTU Observed OTU
UNOISE3 Observed ASV

Supplemental Figure 10: Scatterplots of ASVs/OTUs found in each sample between the different sequence processing methods on the human associated dataset.

Pairwise scatterplots of the number of ASVs/OTUs found in each biological sample with two different sequence processing methods on each axis. The R value represents the rho correlation between the two different processing methods.
