# Supplementary material for: Denoising the Denoisers: an independent evaluation of microbiome sequence error-correction approaches
Source: PeerJ. 2018 Aug 8;6:e5364. doi: 10.7717/peerj.5364 (PMC6087418; doi:10.7717/peerj.5364)
Supplement: Table S1 [file peerj-06-5364-s011.pdf]

Study	Method	Filter	100% Expected	97% Expected	100% Database	97% Database	Unmatched	Total
HMP	Dada	High	25	5	0	1	12	43
HMP	Deblur	High	21	0	0	0	0	21
HMP	Unoise	High	23	3	0	0	2	28
Mock-12	Dada	High	26	2	44	3	1	76
Mock-12	Deblur	High	16	1	11	1	0	29
Mock-12	Unoise	High	17	2	12	4	0	35
Mock-9	Dada	High	10	1	3	2	12	28
Mock-9	Deblur	High	10	0	3	6	8	27
Mock-9	Unoise	High	10	0	3	9	16	38
Zymomock	Dada	High	8	3	0	0	0	11
Zymomock	Deblur	High	8	0	0	0	1	9
Zymomock	Unoise	High	8	11	0	3	5	27
HMP	Dada	Med	25	7	0	0	10	42
HMP	Deblur	Med	21	0	0	1	0	22
HMP	Open	Med	20	176	2	40	215	453
HMP	Unoise	Med	23	3	0	4	4	34
Mock-12	Dada	Med	26	2	46	3	1	78
Mock-12	Deblur	Med	16	1	11	1	0	29
Mock-12	Open	Med	19	815	72	1202	6783	8891
Mock-12	Unoise	Med	17	2	12	6	0	37
Mock-9	Dada	Med	10	1	3	2	13	29
Mock-9	Deblur	Med	10	0	3	6	8	27
Mock-9	Open	Med	10	25	1	10	50	96
Mock-9	Unoise	Med	10	0	3	9	16	38
Zymomock	Dada	Med	8	3	1	0	0	12
Zymomock	Deblur	Med	8	0	0	1	12	21
Zymomock	Open	Med	8	104	4	24	154	294
Zymomock	Unoise	Med	8	15	0	1	19	43
HMP	Dada	Low	25	7	0	0	9	41
HMP	Deblur	Low	21	0	0	0	0	21
HMP	Unoise	Low	23	3	0	4	6	36
Mock-12	Dada	Low	26	2	46	3	1	78
Mock-12	Deblur	Low	16	1	11	1	0	29
Mock-12	Unoise	Low	17	2	12	6	0	37
Mock-9	Dada	Low	10	1	3	2	13	29
Mock-9	Deblur	Low	10	0	3	6	8	27
Mock-9	Unoise	Low	10	0	3	9	16	38
Zymomock	Dada	Low	8	3	1	0	0	12
Zymomock	Deblur	Low	8	0	0	3	12	23
Zymomock	Unoise	Low	8	15	0	1	20	44
