# Supplementary material for: Denoising the Denoisers: an independent evaluation of microbiome sequence error-correction approaches
Source: PeerJ. 2018 Aug 8;6:e5364. doi: 10.7717/peerj.5364 (PMC6087418; doi:10.7717/peerj.5364)
Supplement: Table S2 [file peerj-06-5364-s012.pdf]

Organism	DADA2 Observed Abundance	Deblur Observed Abundance	UNOISE3 Observed Abundance	OTU Observed Abundance	Expected Abundance
Acinetobacter baumannii ATCC 17978	3.054%	3.321%	2.865%	3.207%	5.000%
Actinomyces odontolyticus ATCC 17982	1.764%	1.863%	1.611%	1.800%	5.000%
Bacillus cereus ATCC 10987	3.305%	3.569%	3.621%	3.567%	5.000%
Bacteroides vulgatus ATCC 8482	12.623%	7.566%	11.406%	12.885%	5.000%
Clostridium beijerinckii ATCC 51743	7.926%	7.656%	7.246%	8.031%	5.000%
Deinococcus radiodurans DSM 20539	5.506%	5.743%	4.718%	5.309%	5.000%
Enterococcus faecalis ATCC 47077	2.982%	3.148%	3.172%	3.032%	5.000%
Escherichia coli ATCC 700926	5.039%	5.560%	4.822%	5.279%	5.000%
Helicobacter pylori ATCC 700392	2.867%	3.169%	2.613%	2.946%	5.000%
Lactobacillus gasseri DSM 20243	5.316%	5.728%	5.237%	5.350%	5.000%
Listeria monocytogenes ATCC BAA-679	6.821%	7.265%	7.231%	7.115%	5.000%
Neisseria meningitidis ATCC BAA-335	9.380%	10.267%	8.640%	9.665%	5.000%
Propionibacterium acnes DSM16379	2.661%	2.921%	2.491%	2.796%	5.000%
Pseudomonas aeruginosa ATCC 47085	1.530%	1.200%	1.358%	1.500%	5.000%
Rhodobacter sphaeroides ATCC 17023	3.365%	3.600%	3.044%	0.017%	5.000%
Streptococcus agalactiae ATCC BAA-611	6.078%	6.552%	6.410%	6.422%	5.000%
Streptococcus mutans ATCC 700610	8.466%	9.141%	8.592%	8.697%	5.000%
Streptococcus pneumoniae ATCC BAA-334	2.273%	2.246%	2.323%	2.283%	5.000%
Staphylococcus aureus ATCC BAA-1718/epidermidis ATCC 12228	9.036%	9.480%	9.663%	9.404%	10.000%
Non-Reference	0.009%	0.005%	2.940%	0.698%	0.000%

Supplemental Table 2:

Observed and expected abundances for the Human Microbiome Project mock community.
