# Supplementary material for: Denoising the Denoisers: an independent evaluation of microbiome sequence error-correction approaches
Source: PeerJ. 2018 Aug 8;6:e5364. doi: 10.7717/peerj.5364 (PMC6087418; doi:10.7717/peerj.5364)
Supplement: Table S3 [file peerj-06-5364-s013.pdf]

Organism	DADA2 Observed Abundance	Deblur Observed Abundance	UNOISE3 Observed Abundance	OTU Observed Abundance	Expected Abundance
Bacillus subtilis	8.938%	9.868%	9.061%	8.501%	18.46%
Enterococcus faecalis	2.575%	2.511%	3.154%	2.382%	10.76%
Escherichia coli	16.405%	16.593%	16.396%	18.542%	10.98%
Lactobacillus fermentum	42.975%	44.532%	37.610%	38.308%	15.08%
Listeria monocytogenes	3.850%	4.124%	4.020%	3.854%	15.33%
Pseudomonas aeruginosa	5.001%	4.818%	4.491%	4.656%	5.33%
Salmonella enterica	18.054%	15.549%	18.302%	20.606%	10.86%
Staphylococcus aureus	2.200%	1.871%	2.282%	1.984%	13.64%
Non-Reference	0.002%	0.134%	4.683%	1.167%	0.000%

Supplemental Table 3:

Observed and expected abundances for the Zymomock mock community.
