# Supplementary material for: Denoising the Denoisers: an independent evaluation of microbiome sequence error-correction approaches
Source: PeerJ. 2018 Aug 8;6:e5364. doi: 10.7717/peerj.5364 (PMC6087418; doi:10.7717/peerj.5364)
Supplement: Table S4 [file peerj-06-5364-s014.pdf]

Organism	DADA2 Observed Abundance	Deblur Observed Abundance	UNOISE3 Observed Abundance	OTU Observed Abundance	Expected Abundance
Bacteroides cellulosilyticus DSM 14838.	4.529155%	4.762923%	4.463819%	4.463819%	4.270000%
Bacteroides eggerthii	0.000555%	0.000000%	0.000000%	0.000576%	0.000427%
Bacteroides fragilis	0.030407%	0.000000%	0.000000%	0.007719%	0.042700%
Bacteroides massiliensis	0.005105%	0.000000%	0.000000%	0.006106%	0.004270%
Bacteroides ovatus	46.780400%	52.216690%	46.924230%	45.713600%	42.700000%
Bacteroides thetaiotaomicron	0.045000%	0.000000%	0.000000%	0.017339%	0.042700%
Bacteroides uniformis	0.449948%	0.204068%	0.451612%	0.449141%	0.427000%
Bacteroides vulgatus	37.856410%	33.055450%	38.395760%	37.320940%	42.700000%
Barnesiella intestinihominis	0.000222%	0.000000%	0.000000%	0.000173%	0.000427%
Clostridium celatum JCM 1394	0.040617%	0.039634%	0.040026%	0.039977%	0.042700%
Clostridium cocleatum	0.366716%	0.389210%	0.364273%	0.357550%	0.427000%
Clostridium methylpentusum DSM 5476	0.000000%	0.000000%	0.000000%	0.000000%	0.000427%
Clostridium phytofermentans	0.001276%	0.000000%	0.000000%	0.000691%	0.000427%
Clostridium xylanovorans	3.980113%	4.415016%	3.972042%	3.963583%	4.270000%
Coprococcus comes ATCC 27758	0.305403%	0.342785%	0.305800%	0.304900%	0.427000%
Eubacterium rectale DSM 17629	0.003773%	0.003006%	0.003416%	0.000461%	0.004270%
Howardella ureilytica	0.000444%	0.000000%	0.000000%	0.000461%	0.000427%
Parabacteroides distasonis JCM 13400	2.253178%	2.067511%	2.247355%	4.124471%	0.000427%
Parabacteroides distasonis JCM 13401	2.007813%	1.716153%	1.982890%	0.054839%	4.270000%
Parabacteroides merdae	0.518253%	0.582590%	0.511565%	0.509223%	0.427000%
Parabacteroides sp. D13	0.000000%	0.000000%	0.000000%	0.000288%	0.000427%
Paraprevotella clara YIT 11840	0.004439%	0.003897%	0.004327%	0.004378%	0.004270%
Prevotella buccalis	0.000000%	0.000000%	0.000000%	0.000346%	0.000427%
Prevotella copri DSM 18205	0.000832%	0.000000%	0.000000%	0.000518%	0.000427%
Roseburia intestinalis L1-82	0.004605%	0.003563%	0.004384%	0.007028%	0.004270%
Roseburia inulinivorans DSM 16841	0.036788%	0.044309%	0.036609%	0.036867%	0.042700%
Ruminococcus gnavus ATCC 29149	0.001665%	0.000000%	0.000000%	0.000576%	0.000427%
Non-Reference	0.300891%	0.153190%	0.226546%	2.614085%	0.000000%
