# Supplementary material for: Denoising the Denoisers: an independent evaluation of microbiome sequence error-correction approaches
Source: PeerJ. 2018 Aug 8;6:e5364. doi: 10.7717/peerj.5364 (PMC6087418; doi:10.7717/peerj.5364)
Supplement: Table S5 [file peerj-06-5364-s015.pdf]

Organism	DADA2 Observed Abundance	Deblur Observed Abundance	UNOISE3 Observed Abundance	OTU Observed Abundance	Expected Abundance
Cyberlindnera jadinii	2.246%	2.010%	2.039%	2.039%	6.250%
Debaryomyces hansenii	8.005%	7.929%	7.264%	7.024%	6.250%
Diutina catenulata (*Candida catenulata*)	17.785%	17.906%	16.067%	16.053%	6.250%
Fusarium domesticum	11.347%	10.684%	10.285%	10.262%	6.250%
Galactomyces geotrichum Type 1	5.047%	4.458%	5.490%	4.918%	6.250%
Galactomyces geotrichum Type 2	2.413%	1.558%	2.097%	2.851%	6.250%
Galactomyces geotrichum Type 3	1.290%	5.687%	6.525%	11.381%	6.250%
Galactomyces geotrichum Type 4	5.898%	4.378%	5.342%	1.419%	6.250%
Hyphopichia burtonii	6.516%	6.554%	5.918%	5.915%	6.250%
Kluyveromyces lactis	6.814%	6.853%	6.191%	6.186%	6.250%
Penicillium allii	0.000%	0.000%	0.000%	0.004%	6.250%
Penicillium commune	0.000%	0.000%	0.000%	0.000%	6.250%
Penicillium roqueforti	7.463%	7.493%	6.774%	6.755%	6.250%
Pichia Kudriavzevii	11.932%	11.746%	10.832%	10.814%	6.250%
Scopulariopsis fusca	1.373%	1.363%	1.246%	1.245%	6.250%
Zygosaccharomyces rouxii	1.714%	1.611%	1.557%	1.555%	6.250%
Non-Reference	10.133%	9.747%	12.350%	11.557%	0.000%

Supplemental Table 5:

Observed and expected abundances for the fungal ITS1 mock community.
