# Supplementary material for: Denoising the Denoisers: an independent evaluation of microbiome sequence error-correction approaches
Source: PeerJ. 2018 Aug 8;6:e5364. doi: 10.7717/peerj.5364 (PMC6087418; doi:10.7717/peerj.5364)
Supplement: Table S6 [file peerj-06-5364-s016.pdf]

| Method | Weighted | | | Unweighted | | | Bray-Curtis | | |
|---|---|---|---|---|---|---|---|---|---|
| | DADA2 | UNOISE3 | Deblur | DADA2 | UNOISE3 | Deblur | DADA2 | UNOISE3 | Deblur |
| DADA2 | X | X | X | X | X | X | X | X | X |
| UNOISE3 | 0.904 | X | X | 0.719 | X | X | 0.974 | X | X |
| Deblur | 0.925 | 0.856 | X | 0.772 | 0.835 | X | 0.962 | 0.959 | X |
| OTU | 0.927 | 0.857 | 0.958 | 0.544 | 0.587 | 0.636 | 0.983 | 0.984 | 0.956 |

Supplemental Table 6:

Mantel correlations between distance matrices generated by each method for the Exercise real dataset.
