# Supplementary material for: Denoising the Denoisers: an independent evaluation of microbiome sequence error-correction approaches
Source: PeerJ. 2018 Aug 8;6:e5364. doi: 10.7717/peerj.5364 (PMC6087418; doi:10.7717/peerj.5364)
Supplement: Table S7 [file peerj-06-5364-s017.pdf]

Method	Weighted			Unweighted			Bray-Curtis		
	DADA2	UNOISE3	Deblur	DADA2	UNOISE3	Deblur	DADA2	UNOISE3	Deblur
DADA2	X	X	X	X	X	X	X	X	X
UNOISE3	0.825	X	X	0.663	X	X	0.995	X	X
Deblur	0.962	0.806	X	0.699	0.635	X	0.986	0.982	X
OTU	0.800	0.925	0.764	0.523	0.638	0.590	0.982	0.985	0.971

Supplemental Table 7:

Mantel correlations between distance matrices generated by each method for the human associated real dataset.
