# Supplementary material for: Denoising the Denoisers: an independent evaluation of microbiome sequence error-correction approaches
Source: PeerJ. 2018 Aug 8;6:e5364. doi: 10.7717/peerj.5364 (PMC6087418; doi:10.7717/peerj.5364)
Supplement: Table S8 [file peerj-06-5364-s018.pdf]

Method	Weighted			Unweighted			Bray-Curtis		
	DADA2	UNOISE3	Deblur	DADA2	UNOISE3	Deblur	DADA2	UNOISE3	Deblur
DADA2	X	X	X	X	X	X	X	X	X
UNOISE3	0.970	X	X	0.881	X	X	0.979	X	X
Deblur	0.974	0.976	X	0.917	0.955	X	0.975	0.974	X
OTU	0.975	0.972	0.969	0.863	0.893	0.883	0.974	0.988	0.968

Supplemental Table 8:

Mantel correlations between distance matrices generated by each method for the soil real dataset.
